# Variant Right Renal and Adrenal Arterial Supply With Associated Clinical and Embryological Perspectives

**DOI:** 10.7759/cureus.99558

**Published:** 2025-12-18

**Authors:** Gabriel Mchonde

**Affiliations:** 1 Anatomy and Histology, The University of Dodoma, Dodoma, TZA

**Keywords:** anatomical variations, embryogenesis, inferior adrenal artery, renal artery, retroperitoneal blood supply

## Abstract

Variations in the origin, course, and number of renal and adrenal arteries are common and linked to the embryonic arterial supply of the mesonephros and the ascent of the metanephros. This report aims to document a rare anatomical variant observed in the arterial supply of the right kidney of a 43-year-old formalin-embalmed male cadaver. The right accessory (apical segmental) renal artery originated from the superior mesenteric artery, supplying both the adrenal gland and the apical segment of the right kidney. This case features multiple arterial supplies, along with long primary and secondary prehilar segmental branches. These anatomical variations likely result from the persistence and fusion of lateral splanchnic segmental arteries during embryogenesis. Recognizing such arterial variants in the kidneys and adrenal gland is crucial for radiological and surgical planning, especially in procedures involving retroperitoneal organs.

## Introduction

The kidneys and adrenal glands receive arterial supply from branches of the abdominal aorta, primarily via the renal and adrenal arteries. Classically, a single renal artery arises perpendicularly from the lateral aspects of the abdominal aorta at the level between the first and second lumbar vertebrae [1]. Three adrenal arteries supply the suprarenal glands, which include the superior, arising from the inferior phrenic artery; the middle adrenal artery, arising from the abdominal aorta; and the inferior adrenal artery, arising from the renal artery [2].

Morphological variants in the vascular supply to the kidneys and adrenal glands have been previously reported in origin, course, distribution, and branches [3] and can impact the success of radiological and surgical procedures involving both organs and other retroperitoneal structures. This report presents a case of a triple arterial supply to the right kidney, where the arteries deviated from their typical anatomical origins. Instead, they branched from the superior mesenteric artery and an unusual position on the abdominal aorta. Additionally, a common trunk that emerged from the superior mesenteric artery supplied the adrenal gland and the apical segment of the right kidney. The clinical and embryological implications of this variant are further explored, with a particular focus on the development of the renal and adrenal arterial supply.

## Case presentation

During the routine dissection of the posterior abdominal wall (retroperitoneal) to demonstrate the gross anatomy of the retroperitoneal organs of a 43-year-old, formalin-embalmed male cadaver at the Department of Anatomy, School of Medicine and Dentistry, University of Dodoma in 2024 for medical undergraduate students, a unique renal arterial supply was observed on the right kidney. This cadaver was used following all local and international ethical guidelines and laws on the use of human cadaveric specimens for medical education and research (hence, institutional ethical approval was excluded).

Parietal peritoneum covering the retroperitoneal organs was removed to expose the arteries branching from the abdominal aorta to the kidneys, intestines, and adrenal glands. The right and left kidneys exhibited classic morphology; however, their blood supply demonstrated anatomical variations. Using a Raider Pro stainless steel waterproof digital caliper (resolution: 0.1 mm/0.01-inch, accuracy: ±0.2 mm), measurements were recorded for each relevant vessel under discussion.

Right kidney blood supply

**Figure 1:**
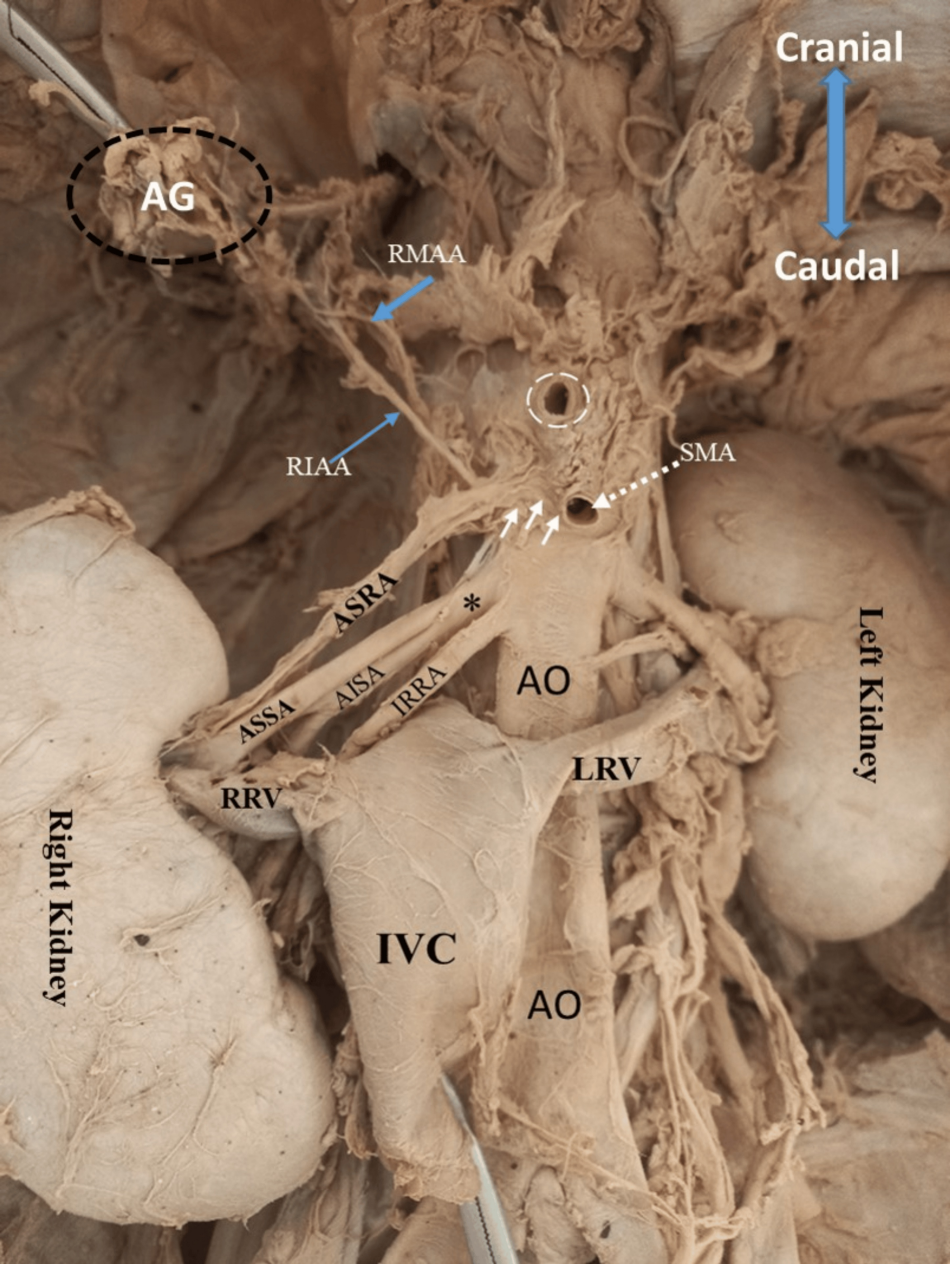
Arterial blood supply to the right kidney and right adrenal gland. A 43-year-old, formalin-embalmed male cadaver showing the retroperitoneal vasculature supplying the kidney and adrenal glands. Note the right kidney is supplied with three renal arteries: apical segmental renal artery (ASRA; white arrowheads), middle right renal artery (asterisk*), and inferior right renal artery (IRRA). The apical segmental renal artery (ASRA; white arrowheads) originates from the superior mesenteric artery (SMA) and gives a right inferior adrenal artery (RIAA) before entering the right kidney. The short middle right renal artery (*) originates from abdominal aorta (AO) and branches to very long anterosuperior segmental artey (ASSA) and anteroinferior segmental artery (AISA). AG: adrenal gland; RMAA: right middle adrenal artery; IVC: inferior vena cava (pulled to display renal arteries); RRV: right renal vein; LRV: left renal vein; white dotted circle: celiac trunk

**Figure 2:**
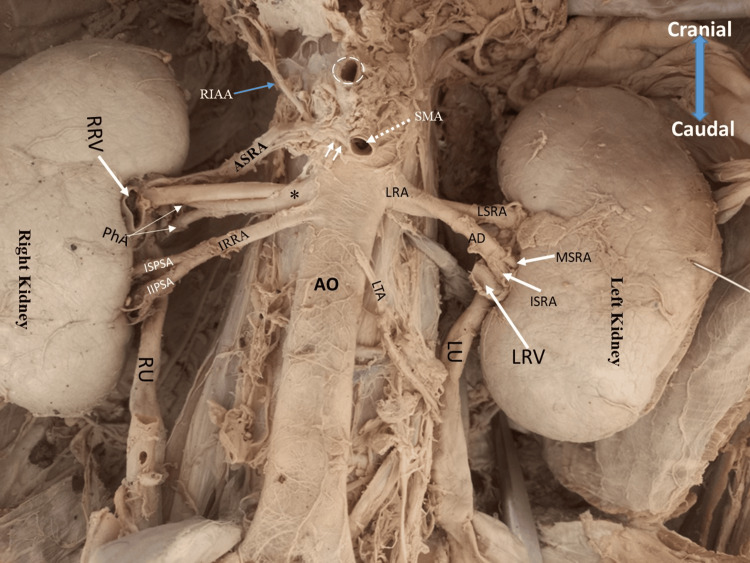
Origin of the renal arteries with their associated segmental/pre-hilar branches. A 43-year-old, formalin-embalmed, male cadaver showing the arterial supply to the kidneys and adrenal glands. Note the multiple arterial supplies to the right kidney with multiple prehilar branches. The left kidney receives supply from the short renal artery (LRA) with very long branches that give secondary divisions as prehilar branches. AD: anterior division of the left renal artery; LSRA: left superior segmental renal artery; MSRA: left middle segmental renal artery; ISRA: left inferior pre-hilar segmental arteries; PhA: pre-hilar arteries; RIAA: right inferior adrenal artery; IRRA: inferior right renal artery; ISPSA: inferior superior pre-hilar segmental artery; IIPSA: inferoinferior pre-hilar segmental artery; RRV: right renal vein (cut); LRV: left renal vein (cut); LTA: left testicular artery; RU: right ureter; LU: left ureter; SMA: superior mesenteric artery; white dotted circle: celiac trunk; *: middle right renal artery

The right kidney received three renal arteries arising from the right lateral aspect of the abdominal aorta: superior, middle, and inferior right renal arteries (Figures 1, 2), which passed posterior to the inferior vena cava.

The superior right renal artery originated as the first branch of the superior mesenteric artery, at the level of the inferior margin of the first lumbar vertebrae, 1 cm below the origin of the celiac trunk. It measured 4.2 cm long with a 4.07 mm external diameter. At a distance of 1 cm from the origin, it gave one branch that ran in a lateral superior direction and supplied the adrenal gland as an inferior adrenal artery, and then continued in the lateral inferior direction to enter the renal hilum on the superior part, which functioned as the superior (apical) segmental renal artery. The inferior adrenal artery measured 3.8 cm long with an external diameter of 1.1 mm.

The middle right renal artery originated 1 cm below the origin of the superior mesenteric artery at the superior margin of the second lumbar vertebrae. At a distance of 1.2 cm, it bifurcated into two branches, giving anterior and posterior divisions, which functioned as the anterosuperior segmental artery and anteroinferior segmental artery due to their anatomical position. The posterior division (anteroinferior segmental artery) ran for 2.3 cm before bifurcating into two pre-hilar branches that entered the middle of the hilum at the posterior aspect (Figures 1, 2). The anterior division (anterosuperior segmental artery) measured 4.2 cm long with an external diameter of 4.4 mm, and entered the kidney at the superior margin of the hilum just posterior to the right renal vein and below the superior (apical) segmental renal artery (Figure 2).

The inferior right renal artery arose from the anterolateral aspect of the abdominal aorta, 0.2 cm below the origin of the middle right renal artery at the inferior margin level of the second lumbar vertebrae. It had an external diameter of 4.44 mm at the point of origin. It bifurcated into superior and inferior pre-hilar segmental branches at a distance of 3.6 cm from its origin. The inferosuperior pre-hilar segmental branch measured 1.2 cm long and entered the hilum at a distance of 0.7 cm below the right renal vein. The inferoinferior pre-hilar segmental branch measured 1.6 cm long and gave two tertiary pre-hilar branches before entering the inferior segment (pole) of the right kidney. Due to its position, the inferior pre-hilar segmental branch functioned as the inferior polar artery of the right kidney.

Right inferior adrenal gland artery

The right inferior adrenal artery originated as the first branch of the superior (apical) renal artery, which is the branch from the superior mesenteric artery (Figures 1, 2), and measured 2.8 cm long with an external diameter of 1.6 mm at the point of origin. It ascended in the superolateral direction to supply the inferior aspect of the right suprarenal gland.

Left kidney blood supply

The left renal artery originated as a single trunk from the lateral aspect of the abdominal aorta at the level between the first and second lumbar vertebrae. It measured 9.23 mm in external diameter at the point of origin and ran left laterally to the left kidney for 0.6 cm before it gave off two branches: anterior and posterior divisions (Figure 2). The posterior division measured 2.3 cm long with an external diameter of 4.2 mm, entered the left renal hilum at the superior margin, and functioned as the left superior hilar artery (Figure 2). The anterior division had an external diameter of 4.3 mm and measured 2.2 cm long (from the point of division) before dividing into two pre-hilar arteries that supplied the middle and inferior part of the left renal hilum (Figure 2); hence, termed as middle and inferior pre-hilar segmental arteries, respectively, based on their anatomical position.

## Discussion

Arterial variations in branching from the abdominal aorta to retroperitoneal organs are common and linked to factors influencing the mechanism of segmental artery development during embryogenesis [4]. Atypical renal artery origins have been reported from the common iliac, superior mesenteric, inferior mesenteric, spermatic, ovarian, or contralateral renal artery [5], while the inferior adrenal gland artery may arise from the renal, abdominal, or renal capsular arteries.

The incidence of triple renal arteries has been reported as 2.5% (ranging from 1% to 4%) in the general population [6]. Additionally, cases of four renal arteries have been documented [7,8]. However, none of these studies have reported the origin of an accessory renal artery from the superior mesenteric artery. An accessory renal artery arising from the superior mesenteric artery and giving rise to the inferior adrenal artery and superior segmental renal artery has not been previously documented. Knowledge about the existence of arterial variants originating anomalously is crucial for preventing accidental injuries to accessory renal arteries and their associated arterial branches during kidney surgeries, such as transplantation. Multiple arterial supply to the kidneys affects renal transplantation by increasing the risk of ischemic insult to the graft. This occurs due to prolonged warm ischemia time, resulting from extended vascular anastomosis.

Embryologically, renal arteries originate from the lateral splanchnic arteries of the dorsal aorta that supply embryonic structures arising from the intermediate mesoderm (nephrogenic mesoderm). Usually, there are several branches of the lateral splanchnic arteries that are associated with three successive kidney systems: pronephros, mesonephros, and metanephros. These arteries are arranged in a ladder-like fashion [9] from the pelvic to the lumbar region. As the successive developmental stages of the kidney ascend on this ladder, the mesonephric arteries degenerate and disappear completely [4], allowing the formation of the new metanephric arteries that become the final renal arteries [2,4]. Perturbations in the molecular signaling pathways and genes controlling endothelial cell behaviors and angiogenic responses may lead to persistent arteries that remain in adults as multiple renal arteries.

Felix [9] noted that as the metanephros ascends, parts of the ladder (lateral splanchnic arteries to the mesonephros and metanephros) persist and develop into the definitive inferior adrenal, inferior phrenic, and renal arteries. This suggests that some of the persistent cranial lateral splanchnic arteries may fuse to give a common trunk of origin for the inferior adrenal and renal artery, as observed in the present case, where the superior right renal artery and inferior adrenal artery had a common trunk.

Wnt/β-catenin signaling pathway has been shown to be involved in cell proliferation, differentiation, and migration during vascular development, while Notch signaling is associated with balancing angiogenic sprouting, vessel stabilization, and vascular maturation [10] through modulation of endothelial cell behavior and angiogenic responses [11,12]. Any mutations or alterations in these molecular players may lead to the development of variants in renal arterial supply, as observed in the present case.

Early branching (pre-hilar branching) of the renal artery is linked with the renal segments, occurring in approximately 10% of the population within 1.5-2.0 cm of the origin of the renal artery [13,14]. In the present case, the branches of renal arteries emerged at a distance of 0.6-1.2 cm from the origin, had a length of 2.3-4.2 cm, and gave two to three secondary branches at the pre-hilar position. The presence of short stems and pre-hilar secondary branches indicates that there could be a persistence and fusion of the lateral splanchnic renal arteries that supplied the mesonephric kidney during the degenerating stage to form the metanephric kidney arteries. Further, the presence of the secondary pre-hilar branches can be explained as a result of alteration in signaling activities such as early or delayed activation between angiogenic factors, such as vascular endothelial growth factor and platelet-derived growth factors, present in the mesenchyme of the blood vessel, and the factors present in the metanephric mesenchyme, such as glial-derived neurotrophic factor and hepatocytic growth factor.

## Conclusions

Variations in the arterial blood supply to the kidney and adrenal gland highlight the complexity of vascular anatomy and its clinical significance. While the renal arteries typically provide primary circulation, accessory renal arteries and diverse adrenal vascular patterns contribute to individual differences. Understanding these variations is essential for surgical interventions, radiological assessments, and managing conditions such as hypertension or adrenal disorders. Recognizing these anatomical differences enhances precision in medical treatments and helps mitigate potential complications in procedures involving the renal and adrenal vasculature and other retroperitoneal organs.
